# Living Donor Liver Transplantation for Intrahepatic Cholangiocarcinoma

**DOI:** 10.3390/curroncol29030157

**Published:** 2022-03-13

**Authors:** Falk Rauchfuß, Aladdin Ali-Deeb, Oliver Rohland, Felix Dondorf, Michael Ardelt, Utz Settmacher

**Affiliations:** Department of General, Visceral and Vascular Surgery, Jena University Hospital, 07747 Jena, Germany; aladdin.ali-deeb@med.uni-jena.de (A.A.-D.); oliver.rohland@med.uni-jena.de (O.R.); felix.dondorf@med.uni-jena.de (F.D.); michael.ardelt@med.uni-jena.de (M.A.); utz.settmacher@med.uni-jena.de (U.S.)

**Keywords:** living donation, malignancy, hepatobiliary disease, transplant oncology

## Abstract

Intrahepatic cholangiocarcinoma is in most transplant regions a contraindication for liver transplantation, even ruling out an active waiting list registration. However, recent studies showed that well-selected patients after a neo-adjuvant treatment benefit from liver transplantation with good long-term outcomes. The role of living donor liver transplantation is unclear for this indication. The current study focuses on LDLT for intrahepatic cholangiocarcinoma.

## 1. Introduction

Intrahepatic cholangiocarcinoma is a rare tumor entity with a poor prognosis. Liver resection plays an important role in the curative treatment of this tumor [1]. In a non-resectable situation, local–regional or systemic approaches might have a positive influence on the oncologic outcome. Liver transplantation has the theoretical advantage to allow surgical radicality but was abandoned in most transplant programs because of poor long-term outcome. The severe organ shortage in most regions worldwide does not allow any expansion of transplantation indication. In the Eurotransplant region, the largest organ allocation institution in Europe, it is regularly not allowed to register a patient with an intrahepatic cholangiocarcinoma if this diagnosis is known at the time of waiting list registration. Liver transplantation with grafts from living donors might be an option to expand the pool of available donor organs but is, especially in Western countries, used with caution. Thus, the proportion of living donor liver transplantation (LDLT) was only 6.7% (Eurotransplant) or 6.16% (United Network for Organ Sharing, UNOS), respectively, based on the total number of performed liver transplantations in the largest allocation system in Europe or North America in 2020.

## 2. What Was the Purpose of This Analysis?

Within this study, we aimed to reflect the role of living donor liver transplantation for intrahepatic cholangiocarcinoma.

We performed a PubMed search using the terms (“intrahepatic cholangiocarcinoma” AND “liver transplantation”), (“intrahepatic cholangiocarcinoma” AND “living donor liver transplantation”) and (“intrahepatic cholangiocarcinoma” AND “transplantation”). Publications dealing with mixed intrahepatic cholangiocarcinoma/hepatocellular carcinoma as well as perihilar cholangiocarcinoma were excluded. Furthermore, incidental cholangiocarcinoma, which were diagnosed in the post-explant histology, or tumors supposed to be a hepatocellular carcinoma were also excluded since the pre-transplant treatment is different. Studies that described liver transplantation with grafts after deceased donation were also excluded from the current study.

Furthermore, we describe two cases from our own department, where we performed LDLT for intrahepatic cholangiocarcinoma:

### 2.1. Case 1

A 58-year-old woman was suspected to have a focal nodular hyperplasia. Due to a significant growth of the tumor in a period of only a few months, malignancy was suspected. The radiological findings were appropriate for an intrahepatic cholangiocarcinoma. A neoadjuvant therapy was not performed. The patient was transplanted with a right living donor lobe from the husband. The tumor stage was pT3, N0, L0, V1, Pn0, R0. CA19-9 was always < 13.0 U/mL. The patient died 232 months after LDLT due to a diffuse gastric carcinoma with peritoneal metastases. A recurrence of the cholangiocarcinoma was never diagnosed.

### 2.2. Case 2

**Figure 1:**
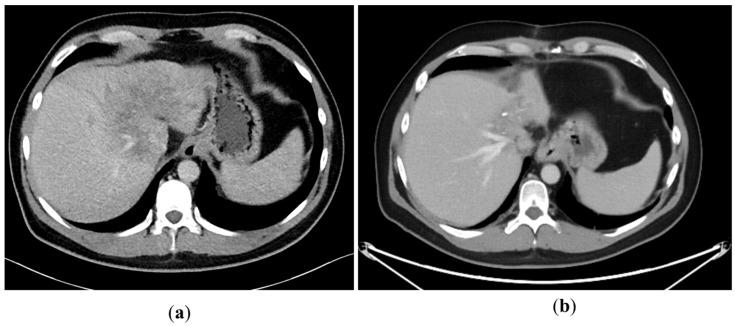
(**a**): Tumor load at the initial presentation of the patient. (**b**) Tumor load after cisplatin/gemcitabine therapy. Note the shrinkage of the left liver lobe.

**Figure 2:**
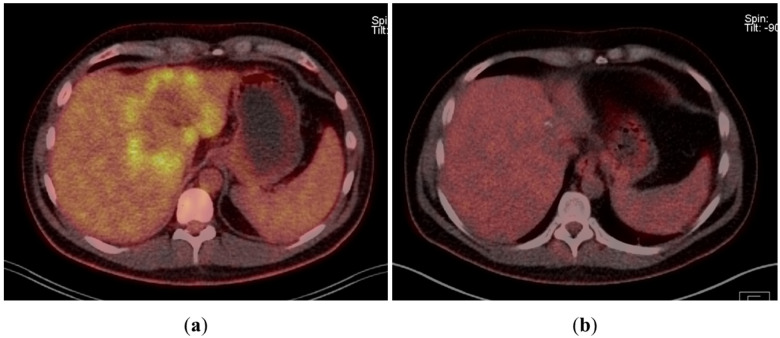
(**a**) Tumor in FDG-PET-CT scan at the time of initial presentation. (**b**) Tumor in FDG-PET-CT scan after chemotherapy and bilobar SIRT.

**Figure 3:**
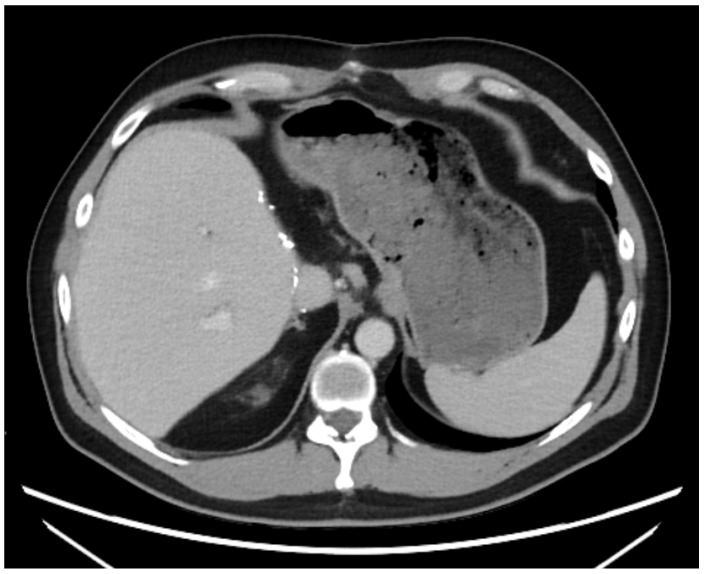
State 15 months after LDLT.

A 43-year-old man went because of persistent fatigue to the general practitioner. The diagnostic showed a large tumor within the central liver (see Figure 1a), which was biopsy-proven as a moderate-differentiated intrahepatic cholangiocarcinoma. An extrahepatic tumor spread could be ruled out (applying an FDG-PET-CT scan, see Figure 2a). Due to the invasion of the hepatic veins, which precluded a primary resection, chemotherapy with cisplatin / gemcitabine was initiated. The tumor showed a partial response (see Figure 1b). We decided to perform surgical exploration with the intent of an ex situ liver resection. Due to an invasion in liver segment VII, even an extended left trisegmentectomy was not possible. We performed an SIRT of both liver lobes resulting in a further, even metabolic (Standardized Uptake Value, SUVmean of the tumor in the initial PET scan: 7.3; SUVmean after treatment: 3.0), response of the tumor (see Figure 2a,b). The CA19-9 levels were always below the reference value. Twenty-two months after the initial diagnosis, the patient could be transplanted with a right living donor lobe from a friend. Histopathologic examination showed an intrahepatic cholangiocarcinoma with a maximum diameter of 6.3 cm. The tumor stage was ypT3, N0, L0, V0, Pn0, R0. Currently, 17 months after the LDLT, the patient is recurrence-free and in good clinical health (see Figure 3). The most recent CA19-9 level is 21.3 U/mL (reference value < 27.0 U/mL).

## 3. Does Liver Transplantation Itself Play a Role in the Treatment of Intrahepatic Cholangiocarcinoma?

Actually, liver transplantation is a contraindication in most countries worldwide, at least if the diagnosis is known at the time point when listing the patients. However, in many studies, which are excluded in the current analysis, the diagnosis of intrahepatic cholangiocarcinoma was made in the post-transplant pathology and most patients were treated as hepatocellular carcinoma. We excluded, as mentioned above, these patients since the bridging therapy differs fundamentally. Another tumor entity which we excluded was mixed hepatocellular cholangiocarcinoma, since the prognosis of these tumors is also different from “pure” cholangiocarcinoma.

However, there are some hints that patients with a non-resectable cholangiocarcinoma might benefit from liver transplantation:

Lunsford et al. [2] described a series of twelve patients who had been evaluated for a liver transplantation. These patients were treated with an extensive neoadjuvant protocol (consisting of a gemcitabine-based chemotherapy or a subsequent second- or third-line regime). The diagnosis of cholangiocarcinoma had to be proven by biopsy or cytology before study inclusion. A key point of this protocol was the response to the neoadjuvant treatment in means of stable disease or regression. Thus, the tumor biology was the most important factor which was addressed with this protocol. Twenty-one patients were screened (whereby nine were subsequently excluded, seven patients due to extrahepatic disease or tumor progression, respectively, and two patients due to a downstaging in a resectable state). From the remaining twelve patients, six were transplanted, three were still on the waiting list and three were not eligible for a transplantation (two because of severe adhesions and one patient was found to be resectable during the exploration). The 1-, 3- and 5-year survival was 100%, 83.3% and 83.3%, respectively. Three patients developed disease recurrence during the follow-up (50%). In this series, two patients were transplanted with domino livers, which is considered as living donation. However, even in the very extensive description of each case, it is not clear which particular patient received the domino organs. Therefore, they were excluded in Table 1. Three other recipients receiving domino grafts were reported by McMillan et al., whereby patient details were again not reproduceable [3].

Sapisochin et al. could retrospectively show that especially patients with “very early” (defined < 2 cm tumor diameter) intrahepatic cholangiocarcinoma have a good prognosis after liver transplantation. However, even the advanced intrahepatic cholangiocarcinoma patients had a 5-year survival rate of 45%. However, 47% of the analyzed tumors were incidental findings in the post-transplant histology [7].

A meta-analysis by Ziogas et al. (2021) reported a 1-, 3- and 5-year survival of 75%, 56% and 42%. The pooled overall recurrence rate was 43%, depending on the tumor size: very early intrahepatic cholangiocarcinoma (<2 cm) had a recurrence rate of 15%, whereas the recurrence rate of advanced cholangiocarcinoma was 51%. The majority of patients suffered from liver cirrhosis (65.6%) [8].

Comparing liver resection and transplantation, there is the huge problem that most studies referring to liver transplantation report either cholangiocarcinoma in liver cirrhosis or incidental cholangiocarcinoma. Both conditions are hard to compare with patients who underwent liver resection since the initial situation is different. If a (major) liver resection is technically possible and suitable from functional liver state, it should be performed as first option. The 5-year survival rate is between 25–40%. Combining these, Mazzaferro et al. recommend that liver transplantation is suitable for selected patients with exclusive liver disease, either in early stages diagnosed in the context of chronic liver diseases or in locally advanced tumors, when neoadjuvant treatments have achieved sustained tumor response without extrahepatic tumor spread [9].

Another factor that should be considered is the immunosuppression needed after a transplantation potentially promoting tumor recurrence or metastases. The group of Lunsford et al. proposed a maintenance therapy with everolimus and tacrolimus [2]. Our patients received a tacrolimus-based regime (Case 1) or a switch to everolimus (Case 2). Due to the low number of cases, it is too early to recommend a distinctive immunosuppressive regime, but it is expectable that mTOR-inhibitors will play a role in it.

## 4. Does LDLT Actual Play a Role in the Treatment of Intrahepatic Cholangiocarcinoma?

There were three studies [4,5,6], plus our two patients, where LDLT for intrahepatic cholangiocarcinoma could be reconstructed, leading to a total number of six patients, where LDLT was performed in a preoperatively known intrahepatic cholangiocarcinoma. The findings are summarized in Table 1. The median follow-up was 30.5 months (range 17–232 months). Two patients died in the reported studies (33%), whereby one patient died 232 months after LDLT without any recurrence. However, it is likely that the patients with recurrent disease died soon after publication of the data. One large study describing LDLT for intrahepatic cholangiocarcinoma had to be excluded since the diagnosis was in all cases incidentally in the post-transplant histology [10]. Another study by Hara et al. dealing with either incidental cholangiocarcinoma or as HCC pre-treated patients showed 1-, 3- and 5-year overall survival rates of 79%, 63%, and 46%, respectively. All patients were treated with LDLT [11]. Vilchez et al. described a series of 440 patients after liver transplantation for intrahepatic cholangiocarcinoma with 16.1% of the patients receiving a LDLT. The 5-year survival of the whole cohort was 47%. However, a detailed description of the LDLT recipients is also missing [12].

Actually, three studies (two in Canada, one in Norway) are recruiting patients with intrahepatic cholangiocarcinoma for liver transplantation. The most relevant in the context of this paper is the study from Toronto (NCT04195503) since this study approach deals with LDLT after a neoadjuvant treatment period. Actually, there is no information available about the state of recruitment. The other two studies (NCT04556214 and NCT02878473) do not require a living donor for study inclusion. Another study, performed in Turkey, deals with mixed HCC/CCC and intrahepatic cholangiocarcinoma which were diagnosed in the post-transplant histology.

Interestingly, a meta-analysis by Ziogas et al. (2021) showed that only 6.6% (14 of 212 recipients) of all liver transplantations for intrahepatic cholangiocarcinoma were performed with LDLT (including the study of Jung et al. with the incidental cholangiocarcinoma) [8], in contrast to the study of Vilchez et al. where the percentage of LDLT for intrahepatic cholangiocarcinoma was even higher [12].

## 5. Is the Donor Risk Reasonable to Justify a New Malignant Entity as a Transplantation Indication?

Dealing with this question, it is necessary to visualize the concept of double equipoise: it means that LDLT should be performed only if the donor risk is justified by the acceptable outcome for the recipient. Since the donor outcome even after right-sided hepatectomy becomes more and more safe, it justifies a 5-year survival of, ideally, 83.3 = %, as reported by Lunsford et al. [2]. Of course, the careful recipient’s selection is sine qua non for the justification of an operation of a healthy person consenting to help as living donor. In some patients, it might be possible to further lower the risk of performing a left-sided partial liver resection as donor procedure, e.g., performing a RAPID (resection and partial liver segment 2–3 transplantation with delayed total hepatectomy) procedure as proposed by Line et al. for colorectal liver metastases [13].

Considering that there is a low median overall survival of patients with advanced cholangiocarcinoma of only 10.3 months [14], LDLT could be a very good option for otherwise non-resectable tumors.

## 6. What Should Be Planned for the Future for Patients with Non-Resectable Intrahepatic Cholangiocarcinoma?

According to the aforementioned data, liver transplantation might be an ideal option for well-selected patients with non-resectable cholangiocarcinoma. Taking the severe organ shortage in most countries into account, LDLT could be the option of choice. The advantages are obvious: the operation is schedulable, and chemotherapy can be interrupted as needed according to the planned operation. Furthermore, the pool of donor organs for “standard indications” is not burdened. The donor risk is assessable. The key criterion prior to transplantation is the response to a (multidisciplinary) neoadjuvant treatment. It is the responsibility of the individual centers to select the best possible recipient to avoid unnecessary harm of a healthy living donor.

Well-selected and neoadjuvant-treated patients with an intrahepatic cholangiocarcinoma might have a similar (good) prognosis to patients with hepatocellular carcinoma beyond the Milan criteria if they are successfully down-staged [15]. The key point in both tumor entities will be the patient’s selection.

## Figures and Tables

**Table 1:** Summary of patients after living donation for intrahepatic cholangiocarcinoma.

Patient Number	Reported by	Recurrent Disease	Underlying Liver Disease	Reported Follow-Up
1	Jonas et al. [4]	Bone and peritoneum	Liver fibrosis	Alive, 31 months
2	Jonas et al. [4]	Bone and lung	Liver fibrosis	Alive, 31 months
3	Takatsuki et al. [5]	No	Caroli’s disease	Alive, 30 months
4	Sotiropoulos et al. [6]	Yes (localization unknown)	Recurrence after extended right-sided liver resection	Dead, 21 months
5	Own data	No	No	Dead, 232 months
6	Own data	No	No	Alive, 17 months

## Data Availability

The data presented in this study are available on request from the corresponding author.
